# Nuclear FGFR1 promotes pancreatic stellate cell-driven invasion through up-regulation of Neuregulin 1

**DOI:** 10.1038/s41388-022-02513-5

**Published:** 2022-11-10

**Authors:** Abigail S. Coetzee, Edward P. Carter, Lucía Rodríguez-Fernández, James Heward, Qiaoying Wang, Saadia A. Karim, Lina Boughetane, Christopher Milton, Firat Uyulur, Jennifer P. Morton, Hemant M. Kocher, Richard P. Grose

**Affiliations:** 1 Centre for Tumour Biology, Barts Cancer Institute, Queen Mary University of London, London, EC1M 6BQ UK; 2 Centre for Cancer Genomics and Computational Biology, Barts Cancer Institute, Queen Mary University of London, London, EC1M 6BQ UK; 3 Cancer Research UK Beatson Institute, Garscube Estate, Switchback Road, Glasgow, G61 1BD UK; 4 Institute of Cancer Sciences, University of Glasgow, Glasgow, G61 1QH UK

**Keywords:** Cancer microenvironment, Pancreatic cancer, Growth factor signalling

## Abstract

Pancreatic stellate cells (PSCs) are key to the treatment-refractory desmoplastic phenotype of pancreatic ductal adenocarcinoma (PDAC) and have received considerable attention as a stromal target for cancer therapy. This approach demands detailed understanding of their pro- and anti-tumourigenic effects. Interrogating PSC-cancer cell interactions in 3D models, we identified nuclear FGFR1 as critical for PSC-led invasion of cancer cells. ChIP-seq analysis of FGFR1 in PSCs revealed a number of FGFR1 interaction sites within the genome, notably *NRG1*, which encodes the ERBB ligand Neuregulin. We show that nuclear FGFR1 regulates transcription of NRG1, which in turn acts in autocrine fashion through an ERBB2/4 heterodimer to promote invasion. In support of this, recombinant NRG1 in 3D model systems rescued the loss of invasion incurred by FGFR inhibition. In vivo we demonstrate that, while FGFR inhibition does not affect the growth of pancreatic tumours in mice, local invasion into the pancreas is reduced. Thus, FGFR and NRG1 may present new stromal targets for PDAC therapy.

## Introduction

Pancreatic ductal adenocarcinoma (PDAC) has a poor prognosis, with a five-year survival rate of less than 10%. Many patients are diagnosed at an advanced stage of disease, when therapeutic options are limited, which contributes to this poor prognosis. Furthermore, PDAC tumours harbour a dense, hypoxic stroma that interferes with drug delivery and reduces the effects of chemotherapy [1–4]. PDAC is therefore a cancer of unmet clinical need and tumour stroma plays an important role in its biology.

Therapeutic targeting of the stroma has gathered significant interest in recent years, leading to multiple clinical trials, including hedgehog pathway inhibition, hyaluronic acid degradation, and a variety of immunotherapies [5–8]. Pancreatic stellate cells (PSCs) form a core cell type of the pancreatic stroma, and become activated in response to tumour development - contributing to the population of cancer associated fibroblasts (CAFs). Activated PSCs enter a cross-talk with cancer cells to induce tumour cell proliferation and invasion, leading to extracellular matrix remodelling and metastatic spread, making these cells an attractive therapeutic target [9]. However, evidence suggests that PSCs also have tumour suppressive roles, with their removal actually enhancing tumour progression [10, 11]. Thus, understanding and targeting the pro-tumoural properties of PSCs, rather than their depletion, is the more attractive approach.

PSCs are present in the healthy pancreas in a quiescent state, becoming activated upon pancreatic injury. Quiescent PSCs store vitamins in lipid droplets, which are lost upon activation [12]. Treatment with all-trans retinoic acid (ATRA) can revert activated PSCs to a quiescent phenotype and improve chemotherapy response. A combination of gemcitabine and ATRA has been shown to be more effective than either therapy alone, decreasing cancer cell survival, PSC activation and tumour burden in 3D in vitro models and the KPC mouse model of PDAC [3], [13]. In the STARPAC phase 1b clinical trial we have demonstrated that a combination of ATRA, as a stromal targeting agent, along with chemotherapy in PDAC patients is not only safe with excellent pharmacokinetic and pharmacodynamics profiles but also appears to successfully target PSCs therapeutically to make chemotherapy more effective [14].

Fibroblast growth factors (FGF) are a family of 18 growth factors that signal through four FGF receptor tyrosine kinases (FGFR) to elicit a range of context-specific biological effects. FGF signalling is critical for a number of developmental processes and dysfunction is common in many malignancies [15]. In addition to canonical RTK signalling, nuclear import of FGFR1 has been reported in a number of physiological contexts [16], including in breast and pancreatic cancers, where it can promote cancer cell invasion and therapy resistance [17, 18]. In PDAC, we have shown that PSC-derived FGF2 can act in an autocrine fashion to stimulate nuclear import of FGFR1 and promote PSC-led invasion [19], pointing towards FGFR inhibition as a potential stromal therapy. In the present work we demonstrate how nuclear FGFR1 in PSCs directs invasion and explore the therapeutic targeting of this receptor in pre-clinical models.

## Results

### Stellate FGFR1 is required for invasion in 3D models of PDAC invasion

**Fig. 1:**
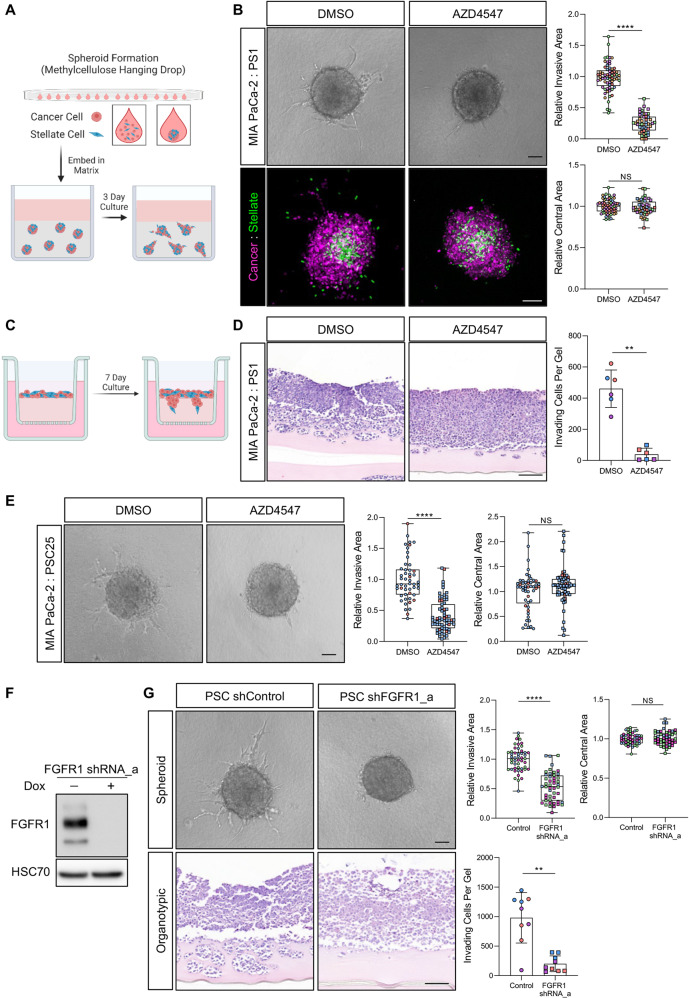
Stellate cell FGFR1 is required for invasion in 3D models of PDAC. **A** Schematic of spheroid model. Cancer cells and PSCs are placed in methylcellulose hanging drops to form spheres. Spheres are subsequently placed in Collagen: Matrigel hydrogels and cultured for 3 days. **B** Brightfield (Top panels) and confocal (Lower panels) images of MIA PaCa-2: PS1 spheres cultured with either DMSO or 1 μM AZD4547 (FGFR inhibitor). MIA PaCa-2 cancer cells are labelled with H2B-RFP (purple) and PS1 PSCs with H2B-GFP (green). Quantification of relative spheroid invasion and central spheroid size also presented. **C** Schematic of organotypic cultures. Cancer cell and PSCs are cultured on top of a Collagen: Matrigel hydrogel and cultured for 7 days. **D** H&E images of MIA PaCa-2: PS1 organotypics cultured with either DMSO or 1 μM AZD4547. Quantification of cell invasion also presented. **E** Brightfield images of MIA PaCa-2: PSC25 spheres cultured with either DMSO or 1 μM AZD4547, presented with relative spheroid invasion and central spheroid area quantification. **F** Western blot of FGFR1 expression in PS1 cells harbouring inducible FGFR1 shRNA_a treated with or without 1 μg/mL doxycycline (Dox) for 48 h. **G** Brightfield (Top panels) and H&E (Lower panels) images of MIA PaCa-2: PS1 spheroids (Top Panels) and organotypics (Lower Panels) with inducible expression of either a control shRNA or FGFR1 shRNA_a in the PSCs. All images representative of at least 3 biological repeats. Individual colours on graphs indicative of technical replicates within each biological replicate. ****P < 0.0001, **P < 0.01, NS Not Significant, Two-tailed T test. Scale bar = 100 μm.

We have previously identified a requirement for FGFR1 in the function of PSCs in PDAC [19]. To explore this further, we developed a novel 3D spheroid model of PSC-led invasion, where cancer cells and PSCs are formed into spheres within methylcellulose-hanging drops then placed into Collagen: Matrigel hydrogels (Fig. 1A). Spheres of MIA PaCa-2 pancreatic cancer cells and PS1 PSCs subsequently exhibit collective invasion. Fluorescently labelled cancer cells and PSCs revealed that, within this model, PSCs act as leaders, followed by pancreatic cancer cells (Fig. 1B).

Inhibition of FGFR kinase activity with a clinically relevant compound, AZD4547 [20], significantly perturbs invasion, without affecting the size of the central sphere (Fig. 1B). This suggests that AZD4547 directly reduces invasion rather than this being a consequence of reduced proliferation. Indeed, AZD4547 has a minimal effect on cell proliferation in a panel of cancer cells and PSCs until high and non-physiological concentrations are administered: something that could not be achieved in humans (Supplementary Fig. 1A). This reduction in invasion was also confirmed in another longer duration, physiomimetic 3D co-culture organotypic model [21], where cancer cells and PSCs are seeded on top of a Collagen: Matrigel hydrogel and allowed to grow and invade over seven days (Fig. 1C, D). This effect of FGFR1 blockade was also replicated when using PDAC-derived primary PSCs, PSC25 and M152 [22], PANC-1 and COLO 357 pancreatic cancer cells (Fig. 1E, Supplementary Fig. 1B–E), and with the chemically distinct FGFR inhibitor PD173074 (Supplementary Fig. 1F), demonstrating relevance across many cancer cell lines as well as CAF subtypes [9].

Next, to address whether the effects of FGFR1 blockade are exclusively PSC-dependent (rather than acting directly on cancer cells, or other FGFRs) we inserted an inducible *FGFR1* shRNA construct into PS1 cells (Fig. 1F, Supplementary Fig. 1G). Knockdown of FGFR1 in just the PSC compartment recapitulated the effects of FGFR inhibition and blocked PSC-led invasion in both our spheroid and organotypic models (Fig. 1G, Supplementary Fig. 1H).

Finally, we examined the effects of inhibiting the main downstream signalling nodes of FGFR1; PKC, ERK, MEK, PI3K, and PLCγ [15]. Inhibition of any of these nodes significantly reduced invasion, with PI3K and PLCγ inhibition having the strongest effect. Inhibition of either MEK or ERK also reduced sphere size, suggesting an additional effect on either cancer cell or PSC proliferation (Supplementary Fig. 1I). Together these data independently validate our previous findings that PSC FGFR1 is exclusively required for effective PSC-led invasion in PDAC [19].

### Nuclear FGFR1 interacts with distinct chromatin regions and induces NRG1 expression

**Fig. 2:**
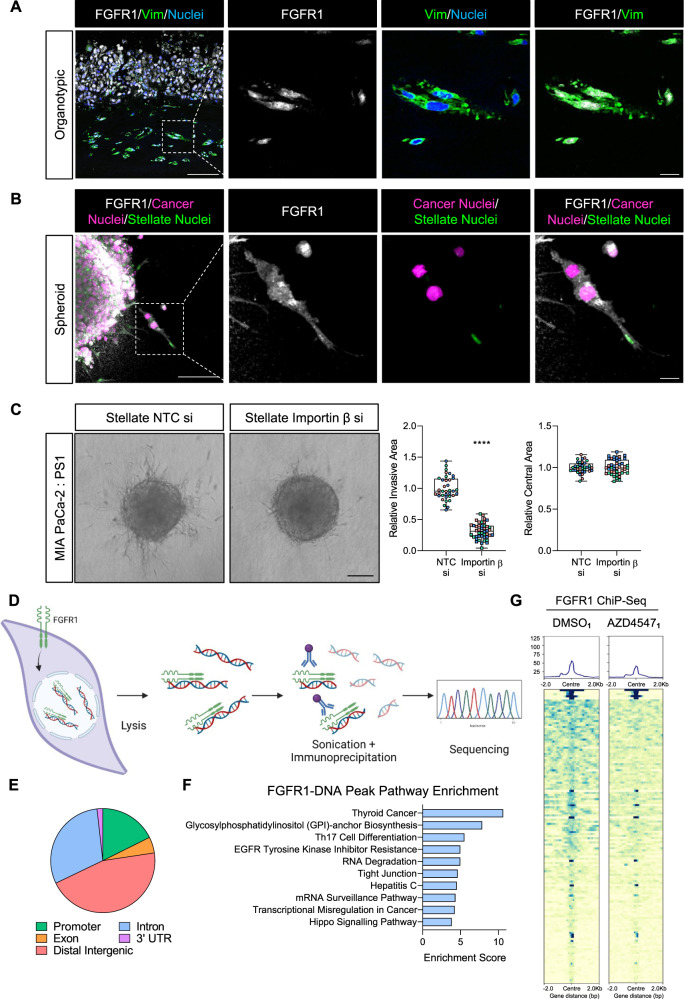
Nuclear FGFR1 interacts with distinct chromatin regions. **A** Representative confocal images of FGFR1 (white) and Vimentin (green) in COLO 357: PS1 organotypic cross sections. **B** Representative immunofluorescence confocal image of FGFR1 expression (white) in MIA PaCa-2: PS1 spheroids. Cancer cells labelled with H2B-RFP (purple), PSCs labelled with H2B-GFP (green). **C** Brightfield images of MIA PaCa-2: PSC25 spheres with PS1-specific knockdown of Importin β. Quantification of relative spheroid invasion and relative central sphere size presented to right of image panels. Individual colours on graphs indicative of technical replicates within each biological replicate. **D** Schematic of FGFR1 ChIP-Seq. **E** Pie chart of FGFR1-DNA binding regions identified within the genome in PS1 cells. **F** Pathway enrichment of FGFR1-DNA binding regions identified in PS1 cells. **G** Heat-map of FGFR1-DNA binding peaks taken from ChIP-Seq data of an individual replicate of PS1 cells treated with either DMSO or 1 μM AZD4547 for 24 h. ****P < 0.0001, Two-tailed T test. Scale bar = 100 μm, =20 μm for insets.

A requirement for FGFR1 nuclear translocation in models of breast and pancreatic invasion as well as in patient samples has been previously reported [17], [19]. In line with these observations we observed nuclear FGFR1 in PSC cell lines and in primary patient derived PSCs/CAFs (Supplementary Fig. 2A, B). Moreover, we observed nuclear FGFR1 specifically in the leading PSCs in our sphere model, and in the invading PSCs in our organotypic model (Fig. 2A, B, Supplementary Fig. 2C, D).

While nuclear FGFR1 is present in PSCs leading invasion, the relative importance of nuclear FGFR1 over canonical RTK signalling for invasion is unclear. To address this we examined PSC-led invasion following PSC knockdown of Importin β, which is required for nuclear transport of FGFR1 [23]. Spheroid invasion was dramatically reduced following Importin β knockdown in the PSC compartment (Fig. 2C, Supplementary Fig. 2E), supporting a requirement for nuclear FGFR in driving PSC-led invasion.

Since nuclear interacting partners for FGFR1 are unknown in this context, we performed ChIP-Seq, immunoprecipitating FGFR1-associated chromatin from PSCs and profiling putative FGFR1 binding sites by sequencing (Fig. 2D, Supplementary Table 1). FGFR1 binding to DNA occurred primarily in distal intergenic regions, with intron and promoter regions forming smaller proportions (Fig. 2E). Pathway analysis of genes associated with FGFR1-DNA binding regions identified enrichment of a number of interesting pathways, including the epidermal growth factor receptor (EGFR) pathway (Fig. 2F). Pre-treatment of PSCs with AZD4547, or depletion of FGFR1 with shRNA, significantly reduced FGFR1-DNA binding, confirming dependence of FGFR1 for isolated peaks (Fig. 2G, Supplementary Fig. 2F, G).

**Fig. 3:**
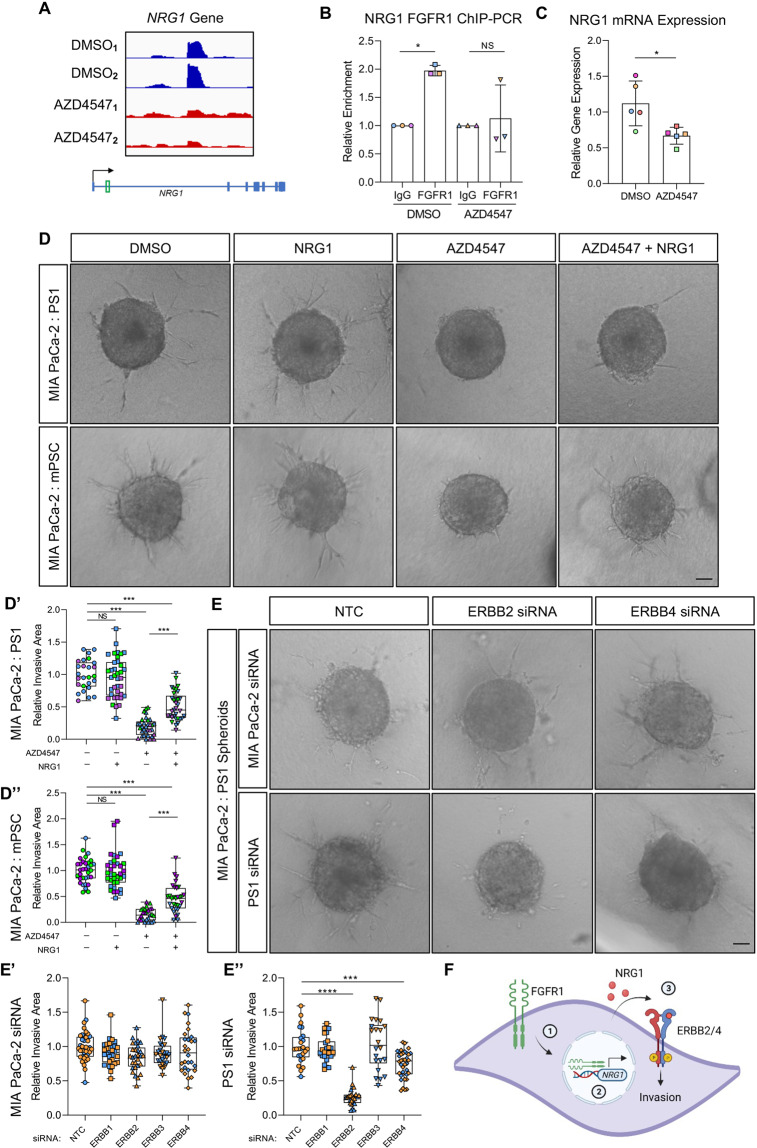
FGFR1 induced NRG1 drives invasion through a HER2/4 autocrine loop. **A** Integrative Genomics Viewer (IGV) snapshot of peaks of FGFR1-DNA binding identified within *NRG1* gene (green box) with and without treatment with 1 μM AZD4547 (FGFR inhibitor). **B** ChIP-PCR of FGFR1 binding to *NRG1* in PS1 cells treated with DMSO or 1 μM AZD4547 for 24 h. **C** *NRG1* expression in PS1 cells treated with DMSO or 1 μM AZD4547 for 24 h. **D** Brightfield images of MIA PaCa-2: PS1 (Top panels) and MIA PaCa-2: mPSC (Lower panels) spheres treated with either recombinant NRG1 (100 ng/mL) or 1 μM AZD4547 for 3 days either alone or in combination. Quantification of relative spheroid invasion presented below image panels (**D’**, **D”**). **E** Brightfield images of MIA PaCa-2: PS1 spheres with either MIA PaCa-2 (Top panels) or PS1 (Lower panels) knockdown of indicated *ERBB* gene. Quantification of relative spheroid invasion presented next to image panels (**E’**, **E”**). **F** Schematic of proposed interaction between FGFR1 and NRG1 in PSCs. FGFR1 translocates to the nucleus where it induces expression of *NRG1*. NRG1 then signals back on PSCs through an ERBB2/4 heterodimer to promote invasion. All images representative of at least 3 biological repeats. Individual colours on graphs indicative of technical replicates within each biological replicate. ****P < 0.0001, ***P < 0.001, *P < 0.05, NS Not Significant, Two-tailed T test or ANOVA with Dunnett’s *post hoc* test. Scale bar = 100 μm.

Given the enrichment of EGFR-related genes and the fact that it was one of the most striking peaks obtained from our FGFR1 ChIP-Seq, we focused on *Neuregulin 1* (*NRG1*). FGFR1 binding was identified in an intron of *NRG1*, with binding lost upon AZD4547 treatment (Fig. 3A, Supplementary Fig. 3A). ChIP-PCR validation confirmed an interaction between FGFR1 and *NRG1* that was AZD4547-dependent (Fig. 3B). This interaction results in NRG1 expression, with AZD4547 repressing *NRG1* transcription in PSCs (Fig. 3C). Thus, FGFR1 can interact with DNA elements within PSCs and regulate gene transcription.

### NRG1 contributes to stellate-led invasion through ERBB2/4

Having identified a dependence on FGFR1 for NRG1 expression, we next questioned whether this contributes to FGFR1-driven invasion. Recombinant NRG1 added to sphere cultures had no significant effect on altering invasion. Strikingly however, when recombinant NRG1 was added to spheres treated with AZD4547, invasion was partially rescued. This was observed in spheres composed of MIA PaCa-2 cancer cells with either PS1 PSCs or mouse PSCs (Fig. 3D, Supplementary Fig. 3B). In further support for a role of PSC NRG1 in mediating invasion, siRNA knockdown of NRG1 in the PSC compartment of spheres significantly reduced invasion (Supplementary Fig. 3C, D).

NRG1 is a ligand for the ERBB (HER) family of RTKs. Inhibition of ERBB activity with Afatinib blocked invasion in spheres, implicating this family in invasion in addition to FGFR1 (Supplementary Fig. 3E). To ascertain the specific receptor and cell type through which NRG1 acts to promote invasion, we performed an RNAi screen of all four ERBB receptors (ERBB1-4) in both the cancer cell and PSC compartments of our sphere model. Invasion was unaffected when any ERBB family member was knocked down in the cancer cell compartment. Conversely, knockdown of either *ERBB2*, or to a lesser extent *ERBB4*, in the PSC compartment, significantly reduced invasion (Fig. 3E, Supplementary Fig. 3F, G). Since ERBB2 has no ligand, instead forming heterodimers with either ERBB3 or ERBB4 to respond to NRG1 [24], based on our data we propose a model where an ERBB2/4 heterodimer signalling mediates NRG1-driven invasion.

Together these data suggest that nuclear FGFR1 in PSCs drives expression of NRG1, which acts in an autocrine loop through an ERBB2/4 heterodimer to promote invasion (Fig. 3F).

### Inhibition of FGFR1 limits invasion in pre-clinical models of PDAC

Having demonstrated a requirement for PSC nuclear FGFR1 in mediating invasion, we next investigated whether FGFR1 inhibitors can be partnered with conventional and upcoming PDAC therapies. We evaluated AZD4547 alongside gemcitabine, a standard chemotherapy for PDAC, and ATRA, a stromal targeting agent that we have previously demonstrated restrains PDAC growth and is currently in clinical trials [3, 14].

**Fig. 4:**
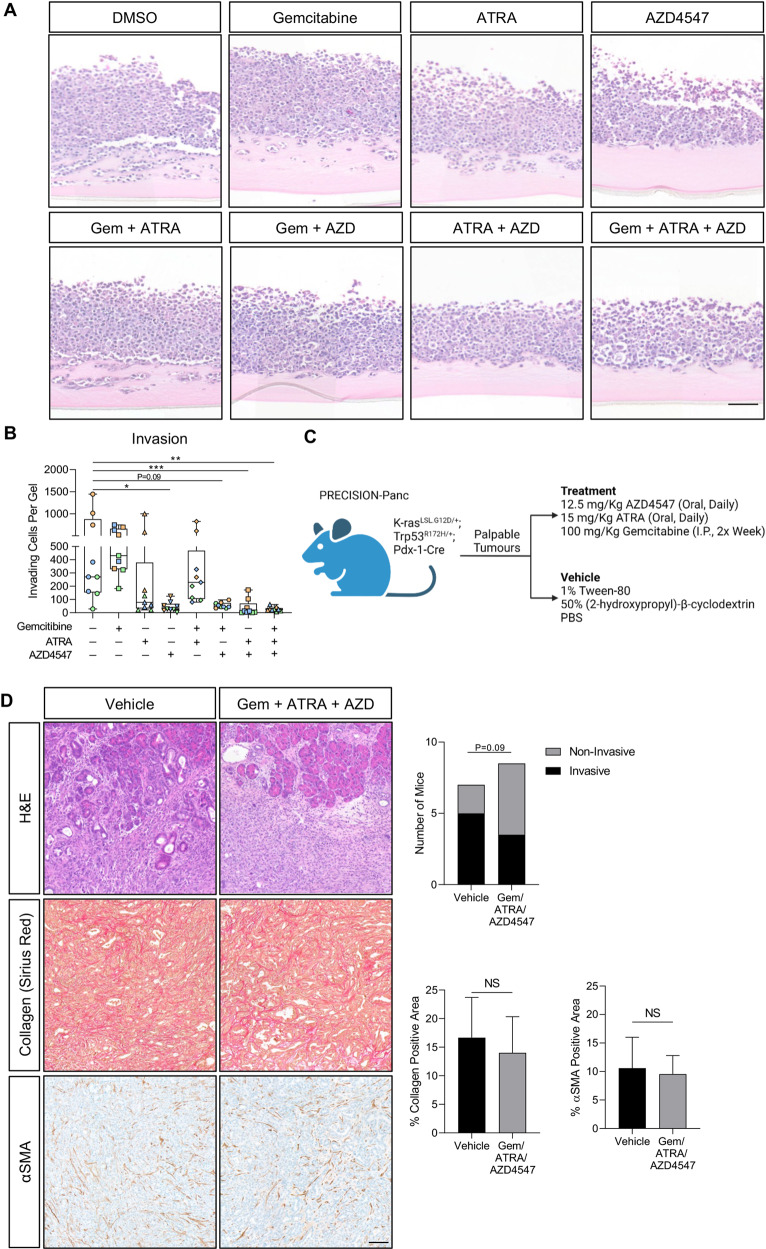
Inhibition of FGFR1 limits invasion in pre-clinical models of PDAC. **A** Representative H&E images of MIA PaCa-2: PS1 organotypics treated with 100 nM Gemcitabine, 1 μM ATRA, or 1 μM AZD4547 either alone or in combination for 7 days. **B** Quantification of invasion from **A**. Images representative of at least 3 biological repeats. Individual colours on graphs indicative of technical replicates within each biological replicate. **C** Schematic of in vivo KPC model and treatment regime. **D** Representative H&E (Top panels), Picrosirius Red (Middle panels), and αSMA IHC (Lower panels) images from KPC mouse pancreatic tumours treated as indicated in **C** (*n* = 7 Vehicle, =8 Gem+ ATRA + AZD). Quantification of invasion (H&E), collagen (Picrosirius Red) and αSMA presented to the right of image panels. ***P < 0.001, **P < 0.01, *P < 0.05, NS Not Significant, ANOVA with Dunnett’s *post hoc* test or Chi Squared test. Scale bar = 100 μm.

Treatment with any agent, either alone or in combination, in 7-day organotypic cultures failed to affect proliferation or apoptosis, measured by Ki67 or cleaved caspase-3, respectively (Supplementary Fig. 4A). However, in all instances AZD4547 treatment, either alone or in combination, demonstrated a significant reduction in invasion, indicating that this effect persists in the presence of additional therapies (Fig. 4A, B).

We next sought to evaluate combination treatment (AZD4547, Gemcitabine, and ATRA) in the KPC mouse model of spontaneous PDAC (Fig. 4C) [25]. Combination treatment was started once mice presented with palpable tumours confirmed by ultrasound. As with organotypic cultures, this combination treatment had little effect on tumour progression and survival (Supplementary Fig. 4B). Moreover, the stromal composition of the tumour was unaltered, with collagen deposition and myofibroblast content, inferred by αSMA staining, unchanged with combination treatment (Fig. 4D). However, pathological analysis of tumours demonstrated a trend for the combination treatment to limit the local invasion of the tumours into the pancreas (Fig. 4D). Combined with data showing only AZD4547-treated organotypics showed reduced invasion, FGFR inhibition is the likely mediator of this in vivo effect.

## Discussion

Stromal targeting in PDAC has received considerable attention and has led to a number of agents entering clinical trials to modulate the stroma and improve chemotherapy efficacy [7, 9]. Understanding the biological mechanisms that allow the stroma to support tumour progression will lead to new-targeted therapies to complement existing strategies.

We previously identified nuclear FGFR1 as a mechanism by which PSCs can facilitate invasion [19], and further demonstrate here the importance for this nuclear RTK in regulating PSC function. Nuclear FGFR1 is also present in breast cancer where it can promote breast cancer cell invasion [17]. More recently ChIP-Seq of nuclear FGFR1 in breast cancer revealed that nuclear FGFR1 can regulate gene transcription and promote resistance to anti-oestrogen therapies [18]. Intriguingly, in this instance it was suggested that nuclear import of FGFR1 was kinase independent, contrary to our findings where treatment with AZD4547, an FGFR kinase inhibitor, significantly reduced FGFR1-DNA binding. Multiple mechanisms for nuclear import of FGFR have been described [16, 17, 26]. FGFR1 can be cleaved by Granzyme B, allowing a truncated form to be trafficked to the nucleus [17]. Alternatively, newly translated FGFR1 can be released from the pre-Golgi membrane into the cytoplasm, where it can interact with FGF2 and ribosomal S6 kinase to facilitate its transport to the nucleus [16]. FGFR1 can also be internalised and transported to the nucleus following stimulation at the plasma membrane [26]. In PSCs, nuclear FGFR1 requires kinase activity and stimulation of the receptor with its ligand FGF2 [19]. Furthermore, here we demonstrate that nuclear import is required for invasion, suggesting that direct canonical FGFR signalling may not be key to the invasive phenotype.

Our ChIP analysis revealed that nuclear FGFR1 regulates *NRG1* transcription. NRG1 then acts in an autocrine fashion in PSCs to promote invasion through an ERBB2/4 heterodimer. Thus, exogenous NRG1 was able to partially rescue the loss of invasion caused by FGFR blockade. This partial rescue likely reflects the additional requirement of canonical RTK signalling downstream of FGFR to facilitate invasion, or additional, currently unexplored, gene regulation by FGFR1. In support of a role for canonical RTK signalling, downstream of FGFR and/or ERBB, inhibition of key RTK signalling nodes abolished invasion in our model systems.

Stromal production of NRG1 has been reported to facilitate progression in several cancers. In prostate cancer, CAF-derived NRG1 can promote resistance to anti-androgen therapy by stimulating ERBB2/ERBB3 heterodimers in cancer cells [27]. A similar paracrine role for stromal-derived NRG1 has been demonstrated in breast cancer [28]. Interestingly, a role for NRG1 in promoting CAF invasion was also observed but was suggested to be through a non-canonical pathway. Tumour cells themselves can also gain NRG1 alterations to promote progression. In PDAC, *NRG1* gene fusions appear to be common in KRAS wild type patients and are sensitive to Afatinib treatment [29].

Our pre-clinical studies were not powered to determine an effect on metastasis and survival. FGFR inhibition appeared to reduce local invasion into the pancreas, but this effect did not reach significance (p = 0.09) and there was no effect on tumour progression. Nevertheless, the strong effect on invasion showcased in our model systems and suggested from our in vivo data suggests that FGFR inhibition may have therapeutic benefit. Other studies have indicated FGFR inhibition may be beneficial in PDAC, either through targeting the stroma, or cancer cell dependent FGFR functions [30]. FGFR1 was amplified in a PDX bone metastasis model of PDAC, which showed reduced tumour growth and bone metastases following treatment with AZD4547 [31]. A further paracrine loop between CAF-derived FGF1 and cancer cell MYC activation has also been identified, with FGFR inhibition showing reduced MYC levels and tumour size, particularly when combined with MEK inhibition [32]. Further pre-clinical studies with an emphasis on metastasis are merited, to demonstrate the utility of FGFR inhibition in PDAC. The addition of ERBB2/4 inhibitors in combination would also be worthy of study, given their role in invasion and tumour progression.

In summary, we have demonstrated a clear mechanism by which nuclear FGFR1 is required for PSC-led invasion in PDAC. We have identified that nuclear FGFR1 regulates the transcription of NRG1, which generates an autocrine loop through ERBB2/4 to further drive invasion. FGFR and/or NRG1 inhibition may present new stromal targets for PDAC therapy.

## Materials and methods

### Inhibitors

GF-109203X (PKC inhibitor), FR180204 (ERK inhibitor), PD0325901 (MEK inhibitor), ZSTK4547 (PI3K inhibitor), U-72122 (PLCγ inhibitor), and AZD4547 (FGFR inhibitor) were all purchased from SelleckChem. PD173074 (FGFR inhibitor) was purchased from Sigma.

### Cell culture

The PDAC cell lines (MIA PaCa-2, PANC-1 and COLO 357) and the stellate cell line (PS1) were cultured as described previously [19]. All cell lines were submitted for short tandem repeat profiling and tested for mycoplasma every six months. The primary cancer-associated stellate cells M152 and PSC25 were isolated and cultured as described [22]. Mouse PSCs were isolated from wild type C57BL/6 mice and cultured as described [33].

### MTS cell viability assay

Cell viability was assessed 72 h after indicated treatments using MTS reagent following manufacturer’s guidelines (G3581, Promega). Absorbance was read at 492 nm, using a 96-well microplate reader (Infinite® F50, Magellan software).

### siRNA transfection

Cells were transfected with siRNA using lipofectamine 3000 (Invitrogen) following manufacturer’s guidelines. SMART Pool siRNAs containing 5 siRNA duplexes targeted against NRG1 (M-004608-02-0005), KPNB1 (M-017523-01-0005), ERBB1 (M-003114-03-0005), ERBB2 (M-003126-04-0005), ERBB3 (M-003127-03-0005), and ERBB4 (M-003128-03-0005) were purchased from Horizon Bioscience.

### Immunostaining

Cells grown on coverslips were fixed and paraffin embedded sections were dewaxed and rehydrated as previously described [19]. Immunohistochemistry was carried out using the Vectastain kit (PK-6101, Vector) and 3, 3’-diaminobenzidine (DAB) (SK-4100, Vector) following manufacturer’s instructions. Spheroid gels were fixed with 4% PFA, permeabilised in 0.1% Triton-X100 and blocked in IF buffer (130 mM NaCl, 7 mM Na_2_HPO_4_, 3.5 mM NaH_2_PO_4_, 7.7 mM NaN_3_, 0.1 % BSA, 0.2 % Triton X-100, 0.05 % Tween-20, 10 % goat serum) for 1 h. Staining was performed with relevant primary antibodies for 48 h at 4 °C and secondary antibodies (1:200) for 2 h at room temperature, diluted in IF buffer (Table 1). Nuclei were counterstained and samples mounted with either Pro-Long® Gold Antifade mountant with DAPI (4, 6-diamidino-2-phenylindole) (P36931, Life Technologies), MOWIOL or Mayer’s haematoxylin (MHS16, Sigma) and DPX (360294H, VWR). H&E and Picosirius red staining was performed by the BCI Pathology Core Services. All slides were viewed using a Pannoramic scanner (3DHISTECH) or a Zeiss LSM Confocal 710 or 880 microscopes (Carl Zeiss MicroImaging LLC). Immunofluorescence images were analysed with ImageJ, while immunohistochemistry images were analysed using Visiopharm (v2019.07.3.7092) and Qupath (v0.3.0) software.

**Table 1:** Antibody conditions.

Antibody	Species	Dilution
Vimentin (M0725, DAKO)	Mouse	1:200 (IF)
FGFR1 (ab10646, Abcam)	Rabbit	1:100 (IF)
FGFR1 (9740, Cell Signalling)	Rabbit	1:500 (WB)
HSC70 (SC-7298, Santa Cruz)	Mouse	1:1000 (WB)
αSMA (M0851, DAKO)	Mouse	1:200 (IHC)
Ki67 (M7240, DAKO)	Mouse	1:100 (IHC)
Cleaved caspase-3 (D175, Cell Signalling)	Rabbit	1:400 (IHC)
Anti-Mouse-HRP (P0447, DAKO)	Goat	1:5000 (WB)
Anti-Rabbit-HRP (P0448, DAKO)	Goat	1:1000 (WB)
Anti-Mouse 488/546 (A11017, A11003, Invitrogen)	Goat	1:500 (IF)
Anti-Rabbit 488/546 (A11034, A11035, Invitrogen)	Goat	1:500 (IF)
Mouse IgG (X0931, DAKO)	Mouse	1:10 (IF)
Rabbit IgG (ab172730, Abcam)	Rabbit	1:100 (IF)

### Western blotting

Cell lysates were prepared using NP40 lysis buffer and denatured proteins separated on 10% SDS-PAGE gels. Gels were run at 140 V for 1.5 h and then transferred onto a nitrocellulose membrane (1060000, GE Healthcare) at 120 V for 1 h. The membranes were blocked in 5% (v/v) milk (70166, Sigma) in 0.1% (v/v) TBST for at least 30 min. Membranes were probed overnight with relevant antibodies (Table 1), then incubated with species relevant HRP-conjugated secondary antibodies before detection using Luminata Forte Western HRP substrate (WBLUF0100, Millipore) and an Amersham Imager 600 (GE Healthcare).

### Generation of inducible cell lines

Two inducible lentiviral shRNA constructs targeted against FGFR1 were created (shRNA_a (CCG-GTG-CCA-CCT-GGA-GCA-TCA-TAA-TCT-CGA-GAT-TAT-GAT-GCT-CCA-GGT-GGC-ATT-TTT) and shRNA_b (CCG-GCC-ACA-GAA-TTG-GAG-GCT-ACA-ACT-CGA-GTT-GTA-GCC-TCC-AAT-TCT-GTG-GTT-TTT) on the tet-pLKO-neo plasmid backbone (#21916, Addgene).

Lentivirus was generated as previously described using H2B-RFP (#26001, Addgene), H2B-GFP (#25999, Addgene), or tet-pLKO-neo (FGFR1 shRNA containing) plasmids [34]. PS1 cells were infected at 30% confluency with viral supernatant and incubated for 24 h. Medium was then replaced and infection efficiency confirmed using the BD FACS Aria Fusion cell sorter (BD Biosciences) or treatment with 600 µg/mL neomycin (shRNA).

### RNA extraction and qPCR analysis

RNA was extracted using the Monarch Total RNA Miniprep Kit (T2010, New England Biolabs) according to manufacturer’s instructions. The RNA was quantified following extraction using a nanodrop ND-1000 spectrophotometer. Reverse transcription was carried out using LunaScript RT SuperMix Kit (E3010, New England Biolabs) according to manufacturer’s instructions. The cDNA was subjected to qPCR analysis using the Luna Universal qPCR Master Mix (M3003, New England Biolabs) with relevant primers (Table 2) and the StepOnePlus Real Time PCR system (ThermoFisher Scientific), according to manufacturer’s instructions.

**Table 2:** PCR Primers.

Target	Sequence – Forward	Sequence – Reverse
ERBB1	TTGCCGCAAAGTGTGTAACG	GTCACCCCTAAATGCCACCG
ERBB2	TGTGACTGCCTGTCCCTACAA	CCAGACCATAGCACACTCGG
ERBB3	GGTGATGGGGAACCTTGAGAT	CTGTCACTTCTCGAATCCACTG
ERBB4	GCAGATGCTACGGACCTTACG	GACACTGAGTAACACATGCTCC
NRG1 (ChIP)	CGCAATCTCGGCTCACTG	CCATCCTGGCTAACAAGGTG
NRG1 (mRNA)	CGTGGAATCAAACGAGATCATCA	GCTTGTCCCAGTGGTGGATGT
Importin β	TGCACTCCTGAACTCATTGG	ACTCGTACCCTCGTATCTGG
Actin	AGAGCTACGAGCTGCCTGAC	AGCACTGTGTTGGCGTACAG

### Mini-organotypic 3D model

Cells were grown in the 3D mini-organotypic model as previously described [21]. Cells were left for 24 h to attach to the gel before treatments were added into the medium below the Transwell insert. At the end of the protocol, gels were washed once in PBS, fixed in formalin for 24 h and washed three times in ethanol before being embedded in paraffin wax.

### Spheroid 3D model

Spheres were formed in 2.5% (v/v) methylcellulose (M0512, Sigma) hanging droplets using 1000 cells total in a 2:1 ratio PSC: cancer cell. Spheres were collected 24 h later and suspended in organotypic mixture (10.5 volumes high concentration Collagen (354249, Corning, 2 mg/mL final concentration), 7 volumes Matrigel, 1 volume HEPES (1 M, pH 7.5, H7006, Sigma) and 21.5 volumes relevant cell culture medium, with 1 M NaOH added dropwise to neutralise the pH), before being seeded into wells of a 96 well plate. Culture medium containing relevant treatments was added on top of gels once set. Gels were imaged using an Axiovert 135 (Carl Zeiss MicroImaging LLC) camera and percentage invasive area quantified using ImageJ (National Institutes of Health), using the following equation: % invasive area = ((total area − central area)/central area) × 100. At the end of the protocol, spheroid gels were washed once in PBS, fixed in 4% PFA for 20 min and washed three times in PBS. Z-stack images of fluorescently labelled spheres were taken using a Zeiss LSM Confocal 880 microscope.

### Chromatin immunoprecipitation

Cells were seeded into 10 ×150 mm dishes per condition to achieve 70–80% confluency at time of harvest (~20 million cells). Cells treated with FGFR inhibitors were harvested 24 h after treatment and shRNA cells were harvested after 48 h of knockdown (doxycycline administration) along with respective contemporaneous vehicle controls and fixed with 1% (v/v) formaldehyde (28908, Thermofisher) in relevant cell culture medium plus protease inhibitor cocktail (05056489001, Roche) on a rocker for 5 min at room temperature. Lysates were quenched with 125 mM glycine solution pH 6.0 (G/0800/60, Fisher Scientific; rocker 5 min, room temperature), washed twice in ice cold PBS and collected by scraping with PBS plus protease inhibitor cocktail. Chromatin was harvested by lysing on ice for 30 min in lysis buffer (1% (v/v) SDS, 50 mM Tris-HCl pH 8.0, 10 mM EDTA plus protease inhibitor cocktail) and sonicated using a Bioruptor pico sonicator (Diagenode, 30 s ON, 30 s OFF for 15 cycles at 4 °C).

Fragmented chromatin was pre-cleared with dynabeads (10003D, Life Sciences, 4 °C, 2 h). After retaining 10% of each sample as an input reference, the remaining chromatin was incubated with 4 µg of anti-FGFR1 antibody or IgG control antibody (Table 1, 4 °C, rotating overnight) with 0.5% (w/v) BSA and 0.1 µg/µL tRNA (10109541001, Roche Diagnostics) to reduce non-specific binding. Samples were then incubated with dynabeads at 4 °C, rotating for 2 h, before going through a series of washes (low salt immune complex, high salt immune complex, LiCl immune complex and TE buffer) and finally eluting in elution buffer at room temperature.

The enriched chromatin samples were then incubated with RNase A (EN0531, Thermo Scientific) and proteinase K (P8107S, New England BioLabs) overnight to remove protein-DNA cross-links. The DNA was extracted from the sample using phenol-chloroform (77617, Sigma) and ethanol precipitation. Qubit and Tapestation analysis confirmed DNA quality and fragmentation before the samples were submitted for sequencing at Oxford Genomics Centre or in-house qPCR analysis. Sequencing hits were analysed by aligning to the reference genome (hs37d5). Reads were mapped using MACS2 and peaks were called using diffBind. Initial analysis of FGFR1 binding was performed by examining enriched peaks in control samples compared to relevant input background control. Known blacklist regions, such as satellite regions, were removed from the analysis. Enriched peaks were then compared between samples to highlight the most reliable hits using Integrated Genome Viewer (IGV). Pathway enrichment of identified peaks conducted using WEB-based GEne SeT AnaLysis Toolkit platform (http://www.webgestalt.org/).

### Animal experiments

All animal experiments were performed under UK Home Office licence and approved by the University of Glasgow Animal Welfare and Ethical Review Board. KPC mice of both sexes were used. Mice were bred in-house at the CRUK Beatson Institute and maintained on a mixed background in conventional caging with environmental enrichment and given access to standard diet and water *ad libitum*. Genotyping was performed by Transnetyx (Cordoba, TN, USA). Mice were monitored at least three times weekly and were randomly assigned treatment groups once palpable tumours emerged and were confirmed by ultrasound imaging. Mice were culled by Schedule 1 method, according to institutional regulations, when exhibiting moderate symptoms of pancreatic cancer (swollen abdomen, loss of body conditioning resembling cachexia, reduced mobility). Statistical assessment of survival from start of treatment was carried out by Kaplan–Meier and Log-Rank analysis.

### Statistical analysis

Apart from analysis of ChIP data all analysis was performed using GraphPad Prism (version 9.0) with relevant statistical tests indicated in relevant figure legend. All data are presented as mean ± SEM.

## Supplementary information

Combined supplementary data

FGFR1 ChIP-seq data
