# Correction: Nuclear FGFR1 promotes pancreatic stellate cell-driven invasion through up-regulation of Neuregulin 1

**DOI:** 10.1038/s41388-022-02557-7

**Published:** 2023-01-20

**Authors:** Abigail S. Coetzee, Edward P. Carter, Lucía Rodríguez-Fernández, James Heward, Qiaoying Wang, Saadia A. Karim, Lina Boughetane, Christopher Milton, Firat Uyulur, Jennifer P. Morton, Hemant M. Kocher, Richard P. Grose

**Affiliations:** 1 Centre for Tumour Biology, Barts Cancer Institute, Queen Mary University of London, London, EC1M 6BQ UK; 2 Centre for Cancer Genomics and Computational Biology, Barts Cancer Institute, Queen Mary University of London, London, EC1M 6BQ UK; 3 Cancer Research UK Beatson Institute, Garscube Estate, Switchback Road, Glasgow, G61 1BD UK; 4 Institute of Cancer Sciences, University of Glasgow, Glasgow, G61 1QH UK

**Keywords:** Cancer microenvironment, Pancreatic cancer, Growth factor signalling

Correction to: *Oncogene* 10.1038/s41388-022-02513-5, published online 10 November 2022

In this article, Lucía Rodríguez-Fernández, James Heward was incorrectly named as one of the contributing authors along with Abigail S. Coetzee, Edward P. Carter. Correct is: Abigail S. Coetzee, Edward P. Carter are contributed equally and Lucía Rodríguez-Fernández, James Heward are contributed equally.

The original article has been corrected.

