# Supplementary material for: Nuclear FGFR1 promotes pancreatic stellate cell-driven invasion through up-regulation of Neuregulin 1
Source: Oncogene. 2022 Nov 10;42(7):491–500. doi: 10.1038/s41388-022-02513-5 (PMC9918430; doi:10.1038/s41388-022-02513-5)
Supplement: Supplementary file 1 — Combined supplementary data [file 41388_2022_2513_MOESM1_ESM.pdf]

Supplementary Figure 1.

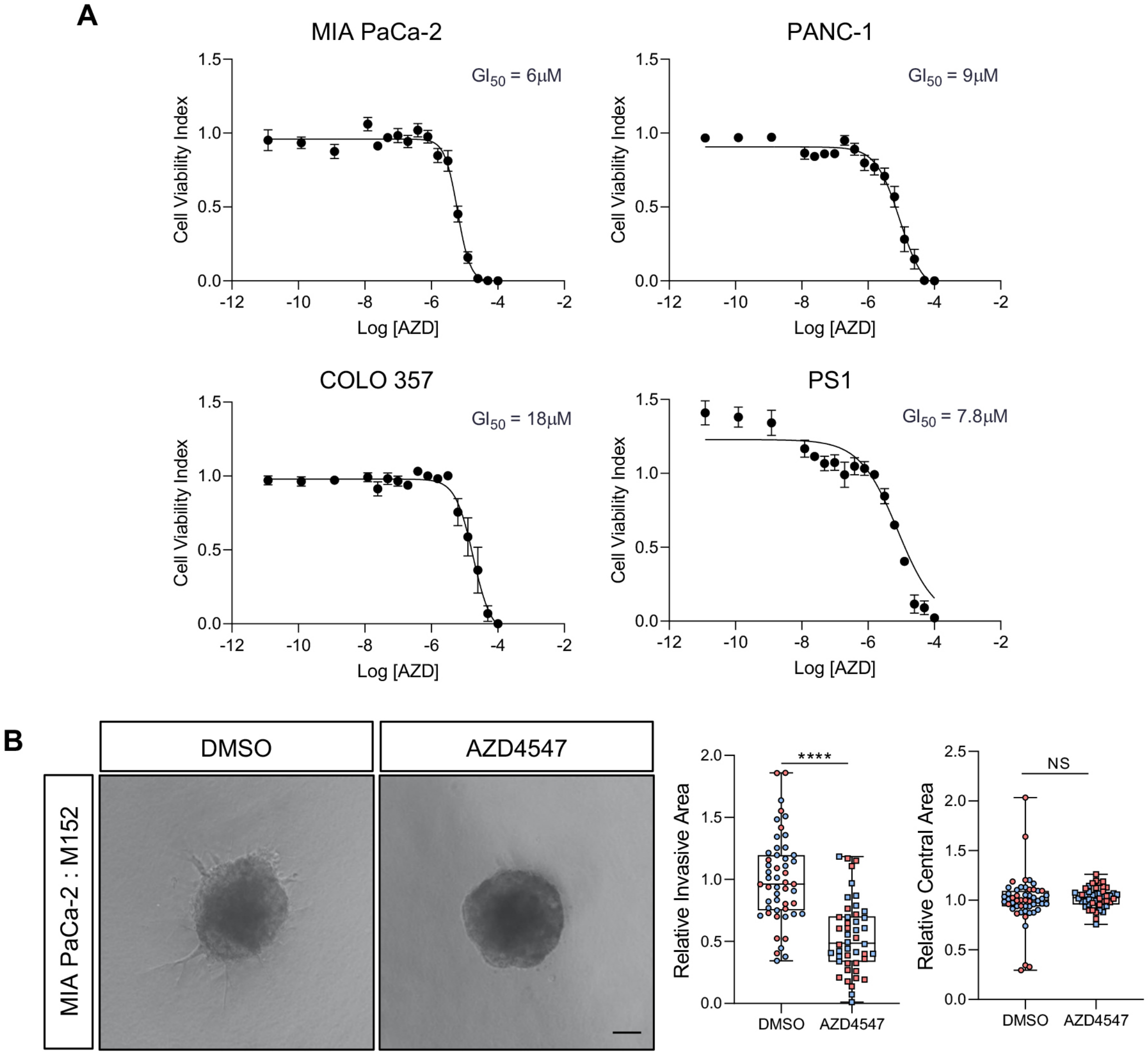

Supplementary Figure 1 (Cont.).

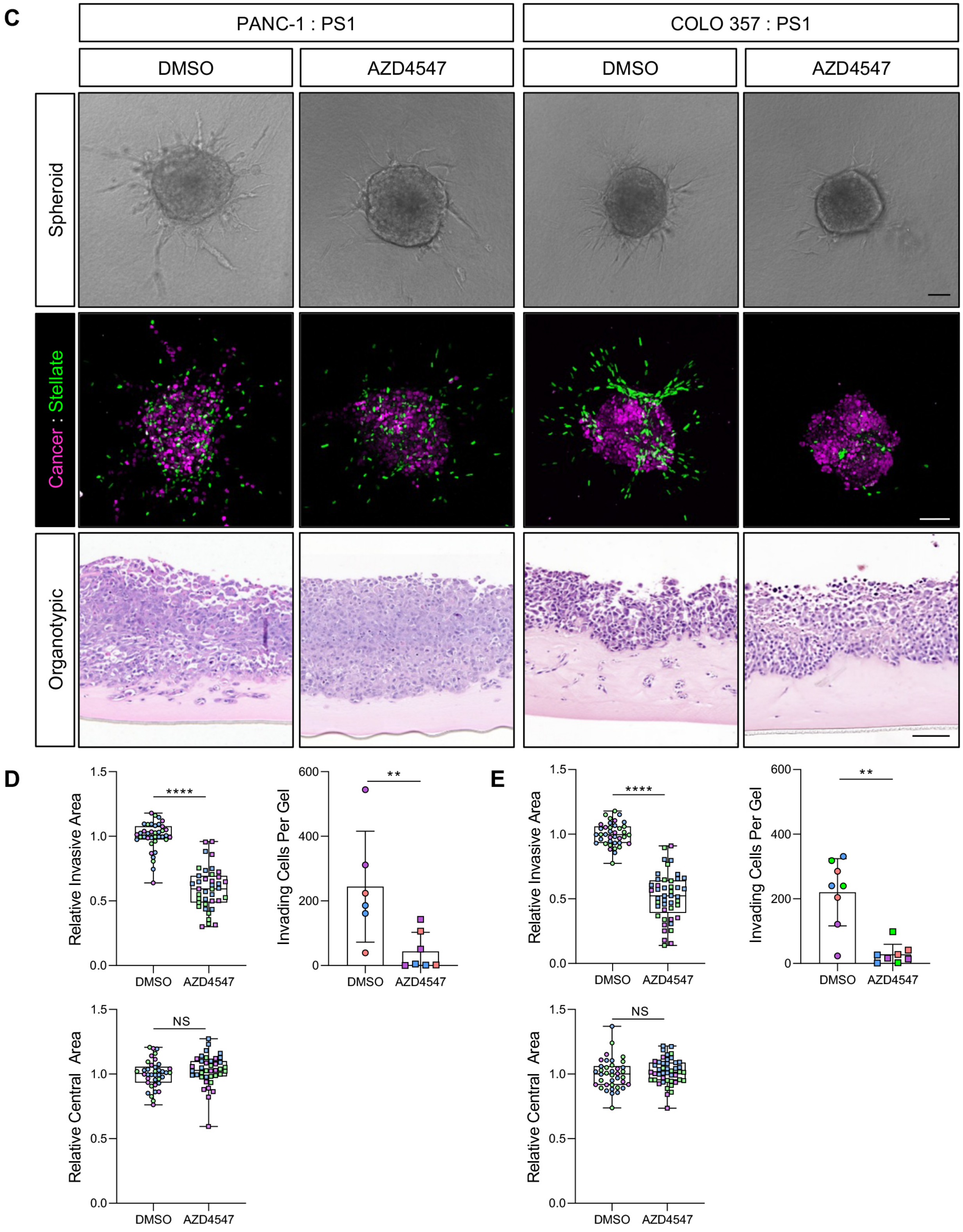

Supplementary Figure 1 (Cont.).

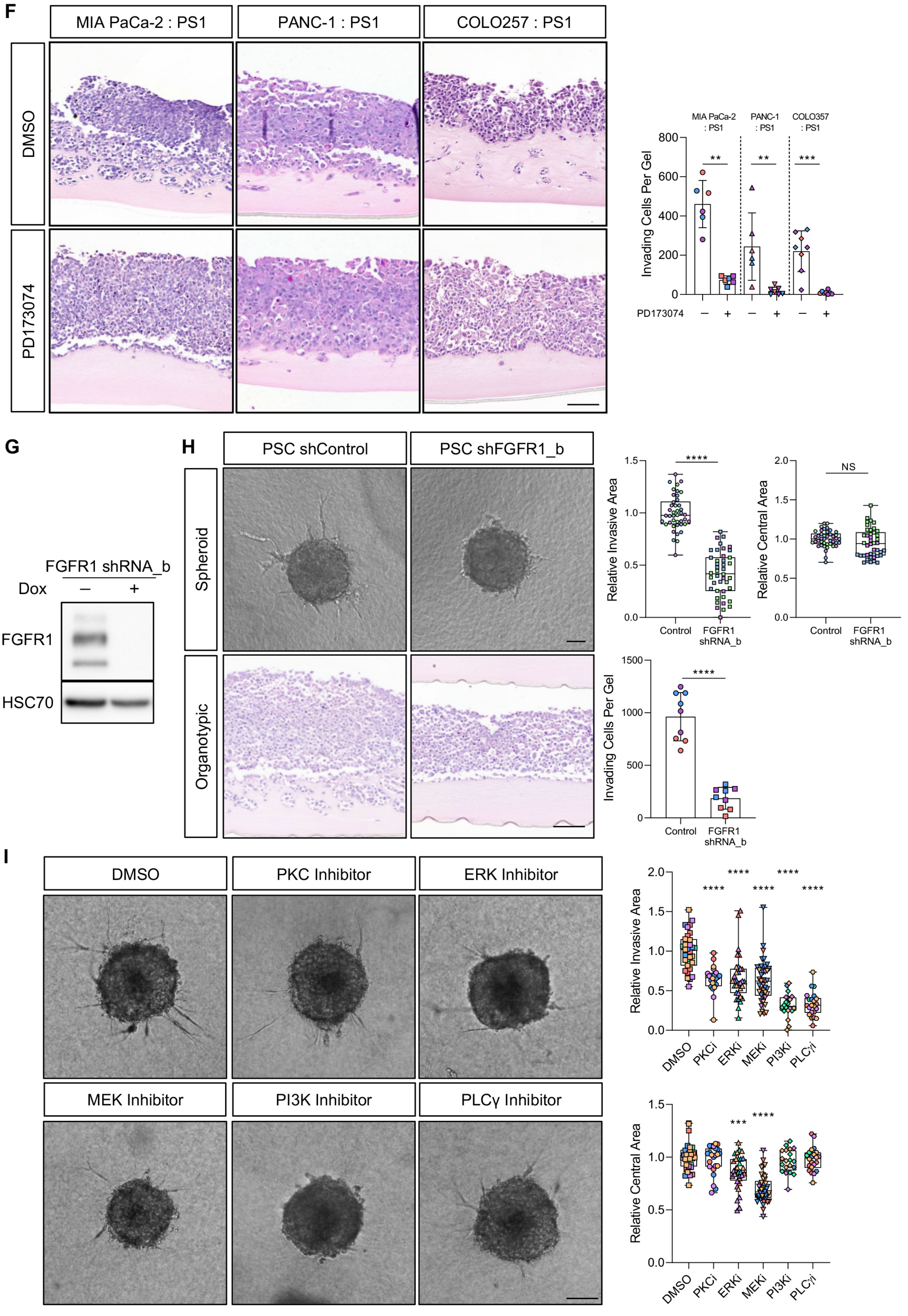

Supplementary Figure 2.

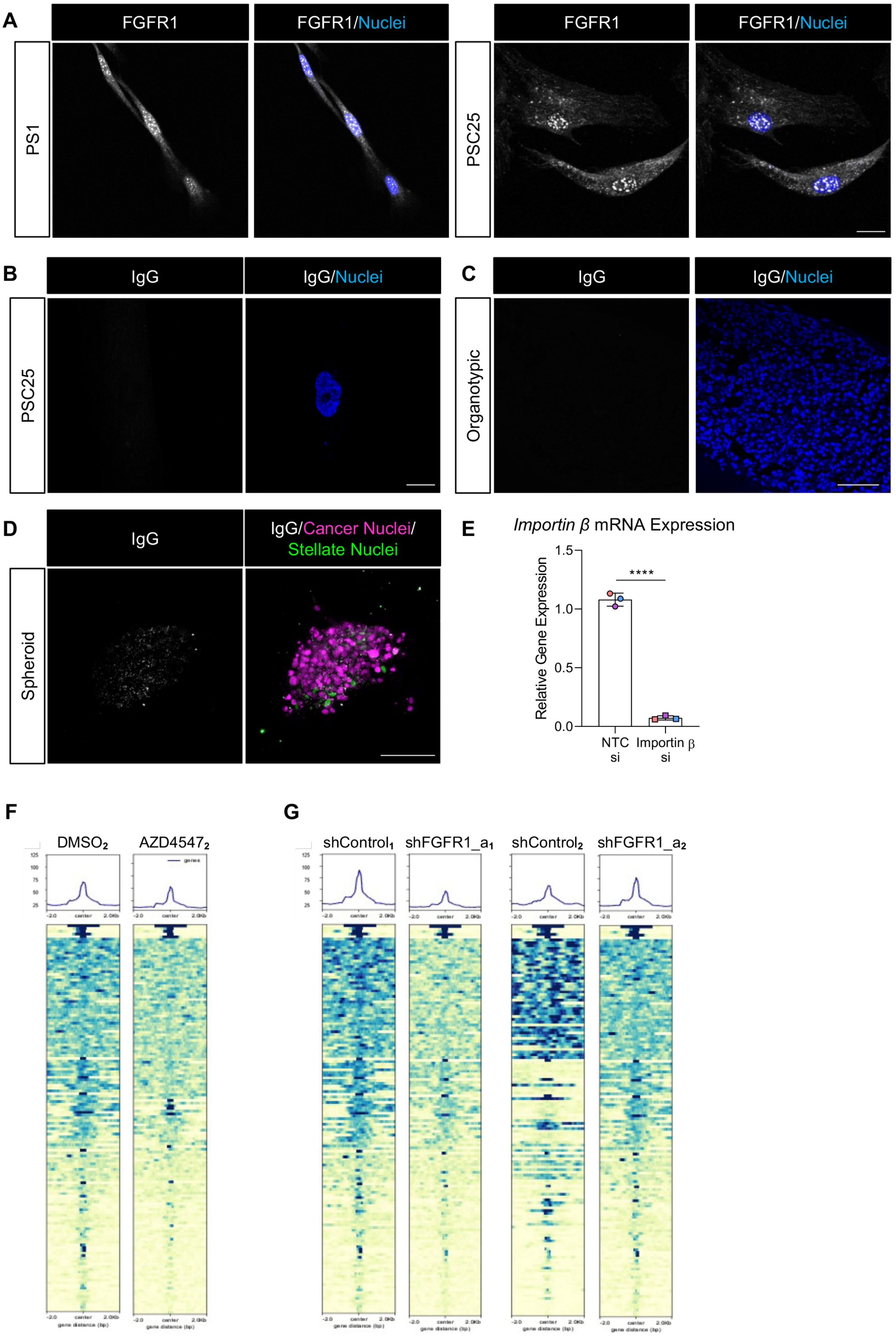

Supplementary Figure 3.

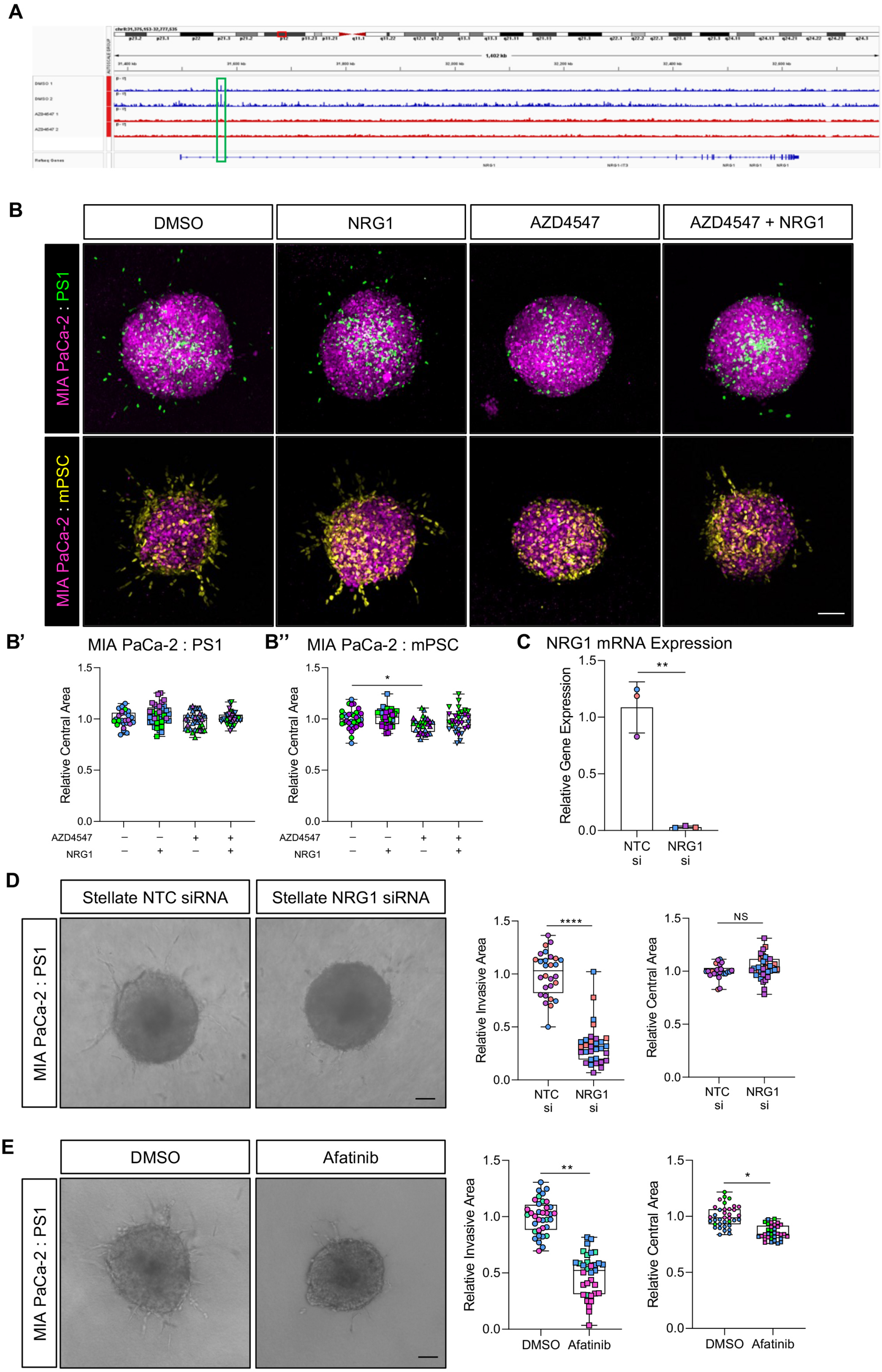

Supplementary Figure 3 (Cont.).

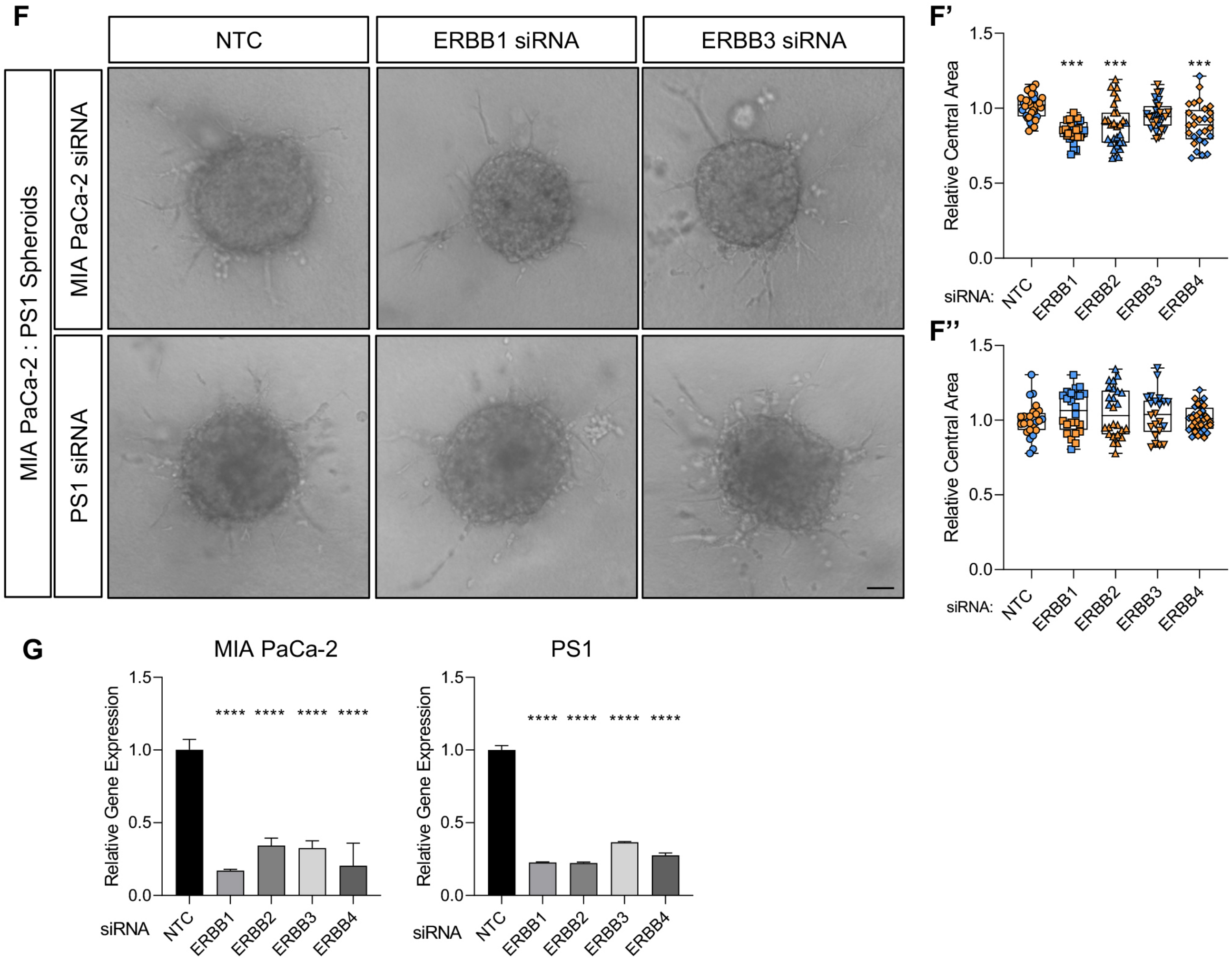

Supplementary Figure 4.

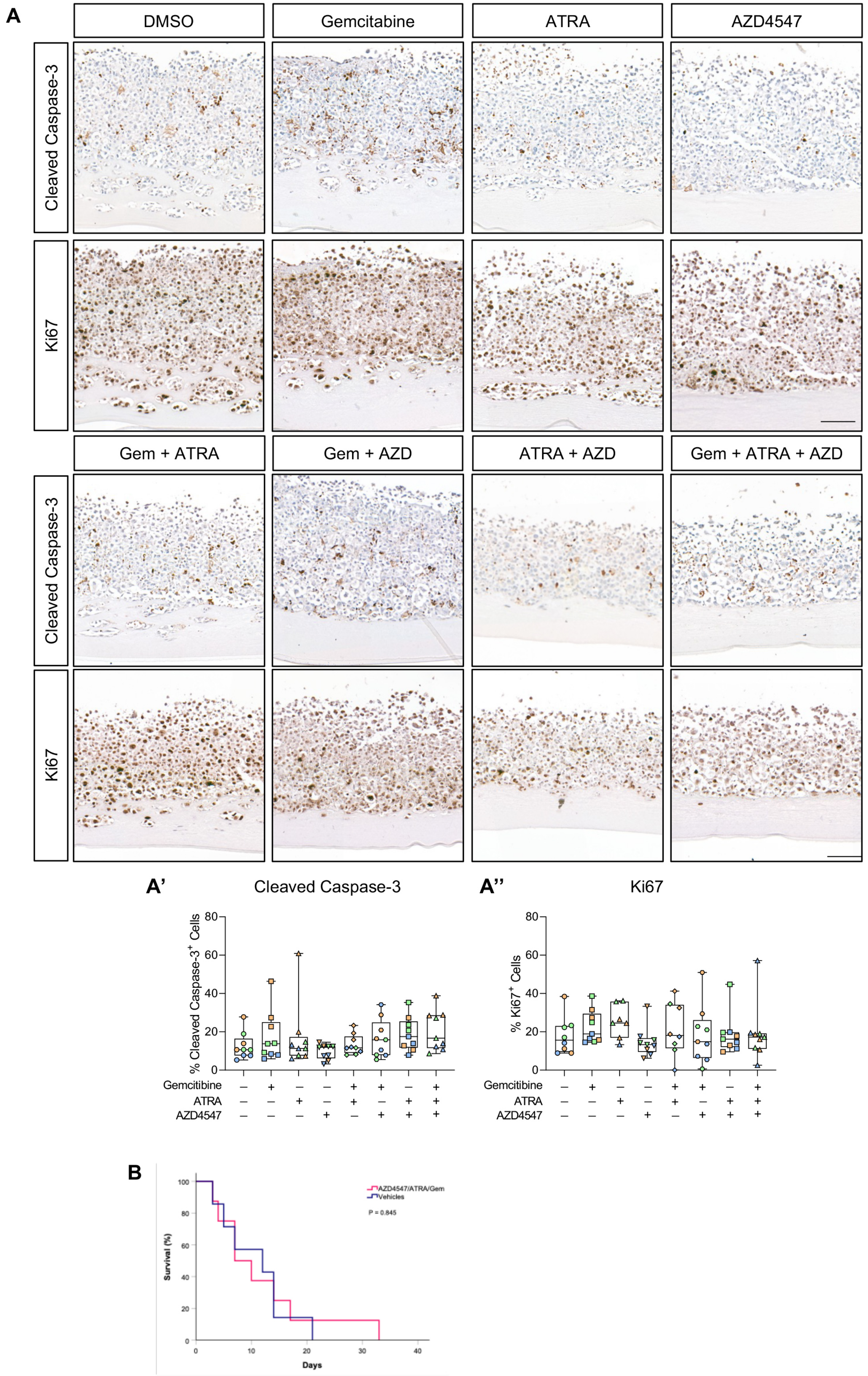

Supplementary Figure 1. A) MTS cell viability of MIA PaCa-2, PANC-1, COLO 357, and PS1 cells following 72-hour treatment with AZD4547 (FGFR inhibitor) at indicated concentrations. B) Brightfield images of MIA PaCa-2: M152 spheres cultured with either DMSO or 1 µM AZD4547, presented with relative invasion and central area quantification. C) Brightfield (Top panels), confocal (Middle panels), and H&E (Lower panels) images of spheroids (Top and Middle Panels) and organotypics (Lower Panels) with either PANC-1 or COLO 357 cancer cells with PS1 PSCs treated with either DMSO or 1 µM AZD4547. Cancer cells are labelled with H2B-RFP (Purple) and PS1 PSCs with H2B-GFP (Green). D & E) Quantification of relative spheroid invasion, central sphere area, and organotypic invasion in PANC-1: PS1 (D) and COLO 357: PS1 (E) cultures. F) H&E of organotypics composed either MIA PaCa-2, PANC-1 or COLO 357 cancer cells cultured with PS1 PSCs treated with either DMSO or 2 µM PD173074 (FGFR inhibitor). Quantification of invasion presented to right of image panels. G) Western blot of FGFR1 expression in PS1 cells harbouring inducible FGFR1 shRNA_b treated with or without 1 µg/mL doxycycline (Dox) for 48 hours. H) Brightfield (Top panels) and H&E (Lower panels) images of MIA PaCa-2: PS1 spheroids (Top Panels) and organotypics (Lower Panels) with inducible expression of either a control shRNA or FGFR1 shRNA_b in the PSCs. Quantification of relative spheroid invasion, central sphere area, and organotypic invasion presented to right of image panels. I) Brightfield images of MIA PaCa-2: PS1 spheroids treated with either DMSO, 5 µM GF-109203X (PKC inhibitor), 40 µM FR180204 (ERK inhibitor), 200 nM PD0325901 (MEK inhibitor), 0.1 µM ZSTK4547 (PI3K inhibitor), or 10 µM U-72122 (PLCγ inhibitor). All images representative of at least 3 biological repeats. Individual colours on graphs indicative of technical replicates within each biological replicate. ****

P<0.0001, *** P<0.001, ** P<0.01, NS Not Significant, Two-tailed T test or ANOVA with Dunnett's post hoc test. Scale bar = 100 μm.

Supplementary Figure 2. A) Representative confocal immunofluorescence images of FGFR expression (white) in PS1 (left panels) and PSC25 (right panels) PSCs. (B-D) Representative IgG primary control confocal images of PSC25 PSCs (B), COLO 357: PS1 organotypic cross-sections (C), and MIA PaCa-2: PS1 spheroids (D). Spheroid cancer cells labelled with H2B-RFP (Purple), PSCs labelled with H2B-GFP (Green). E) Importin β expression following knockdown with Importin β siRNA in PS1 PSCs. (F, G) Heat-map of FGFR1-DNA binding peaks taken from ChIP-Seq data of individual replicates of PS1 cells treated with either DMSO or 1 μM AZD4547 for 24 hours (F), or with and without 1 μg/mL doxycycline (Dox) for 48 hours in FGFR1 shRNA_a PS1 cells (G). Scale bar = 100 μm.

Supplementary Figure 3. A) Integrative Genomics Viewer (IGV) snapshot of peaks of FGFR1-DNA binding identified within NRG1 with and without treatment with 1 μM AZD4547 (Green box). B) Confocal images of MIA PaCa-2: PS1 (Top panels) and MIA PaCa-2: mPSC (Lower panels) spheres treated with either recombinant NRG1 (100 ng/mL) or 1 μM AZD4547 for 3 days either alone or in combination. MIA PaCa-2 cells labelled with H2B-RFP (Purple), PS1 (Green) and mPSC (Yellow) PSCs labelled with H2B-GFP. Quantification of relative central area presented below image panels (B', B''). C) NRG1 expression following knockdown with NRG1 siRNA in PS1 PSCs. D) Brightfield images of MIA PaCa-2: PS1 spheroids with PS1 knockdown of NRG1. Quantification of relative spheroid invasion and relative central sphere size presented to right of image panels. E) Brightfield images of MIA PaCa-2: PS1 spheroids treated

with either DMSO or 1 µM afatinib (EGFR inhibitor). Quantification of relative spheroid invasion and relative central sphere size presented to right of image panels. F) Brightfield images of MIA PaCa-2: PS1 spheres with either MIA PaCa-2 (Top panels) or PS1 (Lower panels) knockdown of indicated ERBB gene. Quantification of relative central area presented next to image panels (F', F''). G) qPCR analysis of indicated ERBB gene knockdown in MIA PaCa-2 and PS1 cells. All data representative of at least 3 biological repeats. Individual colours on graphs indicative of technical replicates within each biological replicate. **** P<0.0001, *** P<0.001, ** P<0.01, NS Not Significant, Two-tailed T test or ANOVA with Dunnett's post hoc test. Scale bar = 100 µm.

Supplementary Figure 4. A) Representative cleaved caspase-3 (Top panels) and Ki67 (Lower panels) IHC images of MIA PaCa-2: PS1 organotypics treated with 100 nM Gemcitabine, 1 µM ATRA, or 1 µM AZD4547 either alone or in combination. Quantification of cleaved caspase-3 (A') and Ki67 (A'') presented below image panels. B) Survival plot of KPC mice treated with vehicle or Gem+ATRA+AZD as indicated in Figure 4C. Images representative of at least 3 biological repeats. Individual colours on graphs indicative of technical replicates within each biological replicate. Scale bar = 100 µm.

Table 1. Antibody Conditions

Antibody	Species	Dilution
Vimentin (M0725, DAKO)	Mouse	1:200 (IF)
FGFR1 (ab10646, Abcam)	Rabbit	1:100 (IF)
FGFR1 (9740, Cell Signalling)	Rabbit	1:500 (WB)
HSC70 (SC-7298, Santa Cruz)	Mouse	1:1000 (WB)
αSMA (M0851, DAKO)	Mouse	1:200 (IHC)
Ki67 (M7240, DAKO)	Mouse	1:100 (IHC)
Cleaved caspase-3 (D175, Cell Signaling)	Rabbit	1:400 (IHC)
Anti-Mouse-HRP (P0447, DAKO)	Goat	1:5000 (WB)
Anti-Rabbit-HRP (P0448, DAKO)	Goat	1:1000 (WB)
Anti-Mouse 488/546 (A11017, A11003, Invitrogen)	Goat	1:500 (IF)
Anti-Rabbit 488/546 (A11034, A11035, Invitrogen)	Goat	1:500 (IF)
Mouse IgG (X0931, DAKO)	Mouse	1:10 (IF)
Rabbit IgG (ab172730, Abcam)	Rabbit	1:100 (IF)

Table 2. PCR Primers

Target	Sequence – Forward	Sequence – Reverse
ERBB1	TTGCCGCAAAGTGTGTAACG	GTCACCCCTAAATGCCACCG
ERBB2	TGTGACTGCCTGTCCCTACAA	CCAGACCATAGCACACTCGG
ERBB3	GGTGATGGGGAACCTTGAGAT	CTGTCACTTCTCGAATCCACTG
ERBB4	GCAGATGCTACGGACCTTACG	GACACTGAGTAACACATGCTCC
NRG1 (ChIP)	CGCAATCTCGGCTCACTG	CCATCCTGGCTAACAAGGTG
NRG1 (mRNA)	CGTGGAATCAAACGAGATCATCA	GCTTGTCCCAGTGGTGGATGT
Importin β	TGCACTCCTGAACTCATTGG	ACTCGTACCCTCGTATCTGG
Actin	AGAGCTACGAGCTGCCTGAC	AGCACTGTGTTGGCGTACAG
